# Effects of serum albumin on the photophysical characteristics of synthetic and endogenous protoporphyrin IX

**DOI:** 10.1590/1414-431X2022e12272

**Published:** 2022-10-03

**Authors:** D.C.K. Codognato, F.S. Pena, E.R. dos Reis, A.P. Ramos, I.E. Borissevitch

**Affiliations:** 1 Departamento de Física, Faculdade de Filosofia, Ciências e Letras de Ribeirão Preto, Universidade de São Paulo, Ribeirão Preto, SP, Brasil; 2 EcoFarm Alimentando Vidas, Caconde, SP, Brasil; 3 Laboratório de Laser, Centro Experimental de Medicina e Cirurgia, Faculdade de Ciências Médicas, Universidade Estadual de Campinas, Campinas, SP, Brasil; 4 Departamento de Química, Faculdade de Filosofia, Ciências e Letras de Ribeirão Preto, Universidade de São Paulo, Ribeirão Preto, SP, Brasil

**Keywords:** Synthetic and endogenous protoporphyrin IX, Aggregation, Bovine serum albumin, Binding, pH effects

## Abstract

The study of the interaction of synthetic protoporphyrin IX (PpIXs) and protoporphyrin IX extracted from Harderian glands of *ssp Rattus novergicus albinus* rats (PpIXe) with bovine serum albumin (BSA) was conducted in water at pH 7.3 and pH 4.5 by optical absorption and fluorescence spectroscopies. PpIXs is present as H- and J-aggregates in equilibrium with themselves and with monomers. The PpIXs charge is 2^−^ at pH 7.3 and 1^−^ at pH 4.5. This increases its aggregation at pH 4.5 and shifts the equilibrium in favor of J-aggregates. In spite of electrostatic attraction at pH 4.5, where BSA is positive, the binding constant (*K*_b_) of PpIXs to BSA is 20% less than that at pH 7.3, where BSA is negative. This occurs because higher aggregation of PpIXs at pH 4.5 reduces the observed *K*_b_ value. At both pHs, water-soluble PpIXe exists in the monomeric form with the charge of 1^−^ and its *K*_b_ exceeds that of PpIXs. At pH 4.5, its *K*_b_ is 12 times higher than that at pH 7.3 due to electrostatic attraction between the positively charged BSA and the negatively charged PpIXe. The higher probability of PpIXe binding to BSA makes PpIXe more promising as a fluorescence probe for fluorescence diagnostics and as a photosensitizer for photodynamic therapy. The existence of PpIXe in the monomeric form can explain its faster cell internalization. Aggregation reduces quantum yields and lifetimes of the PpIXs excited states, which explains higher phototoxicity of PpIXe toward malignant cells compared with PpIXs.

## Introduction

**Figure 1:**
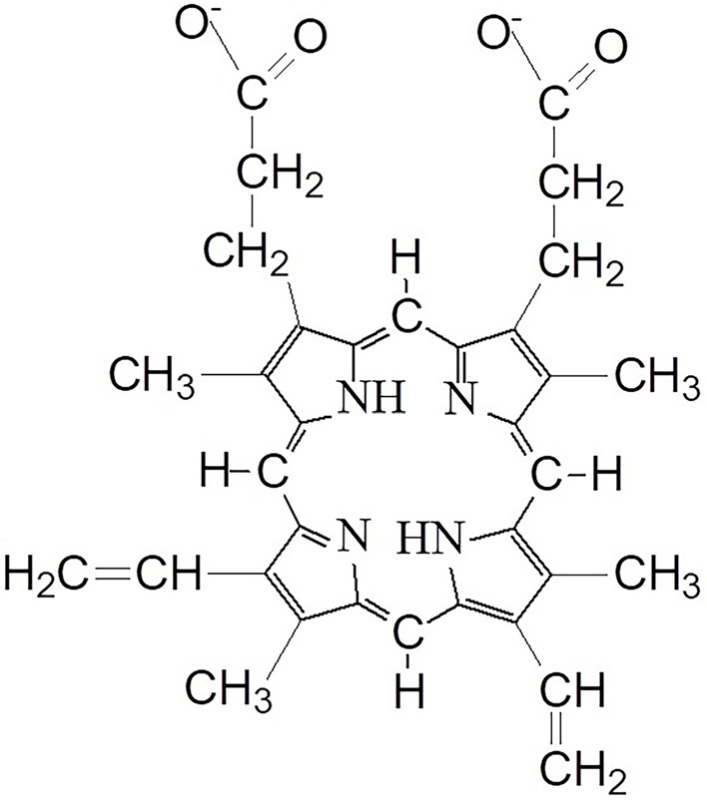
Chemical structure of synthetic protoporphyrin IX.

Protoporphyrin IX (PpIX, Figure 1) is an intrinsic compound of living organisms that plays an important role as a precursor of heme synthesis (1). On the other hand, PpIX is attracting special attention as a photosensitizer (PS) in photodynamic therapy (PDT), a non-invasive method for the treatment of cancer and other diseases, including bacterial and viral infections, and as a fluorescence probe in fluorescence cancer diagnostics (FD) (2,3). The interest in PpIX as a PS has significantly increased after development of the ALA technique, which is based on the introduction of δ-aminolevulinic acid (ALA) into an organism that induces PpIX over-expression in treated tissues (4).

Both synthetic and native PpIX have already been applied in PDT and in FD (5–8). In a living organism, PpIX appears in a specific environment, which includes various nanoorganized structures, such as nucleic acids, proteins, cell membranes, etc. Interaction with these structures can affect the efficiency and even the action mechanism of both synthetic and native PpIX in different ways (9,10). Therefore, the comparison of the interaction of synthetic and native PpIX with nanoorganized structures is of great importance, since it allows to understand their behavior in the organism. PpIX extracted from the Harderian glands of *ssp Rattus norvegicus albinus* rats (PpIX endogenous, PpIXe) is an adequate model of the native type (11). We have demonstrated that PpIXe possesses photodynamic activity toward Harderian gland tissue (12) and murine B16F-10 melanoma cells (13). Moreover, its phototoxicity is 10 times higher and its rate of internalization into the cell is 8-fold higher compared with the synthetic one (13). This makes PpIXe not only an adequate model but also a promising PS for PDT and a fluorescence probe for FD.

Among the various natural nanostructures, serum albumins are of particular interest because they can bind various compounds and transport them with the blood flow in the organism (14). Moreover, the high affinity of albumins for malignant tissues makes them a promising drug delivery system (15,16). On the other hand, PS binding to albumins changes their photophysical characteristics (17–19), which can modify their efficacy in photomedical applications. Characteristics of the environment, such as pH, can affect the interaction between PSs and albumins due to changes in their charge states and/or conformations (20–25); therefore, the effect of pH should be taken into account.

To describe the behavior of porphyrins in water we have to mention the process of aggregation, which is typical even for water-soluble types (18), and references therein). Two types of aggregates can be formed: H-aggregates or face-to-face aggregates and J-aggregates or edge-to-edge ones, which are able to transform into another one and/or be in a dynamic equilibrium (26). H-aggregates are characterized by a blue shift of the optical absorption spectrum compared with that of the respective monomer, while J-aggregates are characterized by the red shift of the absorption spectrum (27). Besides the spectral changes, aggregation reduces dramatically the quantum yield and lifetime of the porphyrin fluorescence and the excited triplet state (28). Therefore, aggregation should be taken into account since it reduces the efficacy of porphyrin application in PDT and FD.

In this research, we studied the binding of synthetic PpIX and PpIX extracted from Harderian glands of *ssp Rattus norvegicus albinus* rats with bovine serum albumin (BSA). The main goal was to establish how the interaction with albumin affects the spectral and kinetic characteristics of the singlet excited state of two protoporphyrins, synthetic (PpIXs) and endogenous (PpIXe), and the effect of their structures upon this interaction. This information is important for the application of these photosensitizers in PDT and FD. The effect of binding on the optical absorption and fluorescence characteristics was studied as a function of the BSA and PpIX concentrations and the pH of solutions.

## Material and Methods

PpIXs and BSA were obtained from Sigma-Aldrich Corporation (USA). PpIXe was extracted from Harderian glands of *ssp Rattus norvegicus albinus* rats in accordance with the procedure described in detail elsewhere (29,30).

To prepare stock solutions, PpIXs and PpIXe were dissolved in a 3/1 mixture of dimethyl sulfoxide (DMSO) and acetonitrile (ACN), both HPLC of purity grade. The experiments were carried out at pH 7.3 in phosphate-buffered saline (PBS) (ionic strength 0.0075 M, 0.005 M NaH_2_PO_4_ + 0.0025 M Na_2_HPO_4_) and at pH 4.5 in acetate (HAc) buffer (ionic strength 0.045 M, 0.02 M NaCH_3_COO + 0.025 M CH?COOH), both prepared with deionized water from a Milli-Q^®^ system. For experiments, the PpIXs and PpIXe stock solutions were diluted in the respective buffers, so that the content of DMSO/ACN mixture was less than 5%. The BSA aqueous stock solution was added to the respective buffer solutions.

Optical absorption spectra were monitored with Beckman Coulter DU-640 spectrophotometer (USA) and the fluorescence spectra were registered by a Hitachi 7000 fluorimeter (Japan).

The time-resolved fluorescence experiments were made using the time-correlated single photon counting technique. The excitation source was a pulse titanium-sapphire laser Tsunami 3950 (USA) pumped by a MillenaXs laser (USA) with 5 ps pulse width at half height and 8.0 MHz frequency, controlled by the pulse picker 3980, all from Spectra Physics (USA). The excitation wavelength was 430 nm obtained by the BBO crystal (GWN-23PL from Spectra Physics). The fluorescence measurements were made with a FL9000 spectrometer from Edinburgh (The Netherlands), adjusted in an ‘L’ configuration with the source of excitation. The measurement wavelength was selected by a monochromator and the detection was made by the Hamamatsu R3809U (Japan) photomultiplier. The average time response of the instrument was ≅ 100 ps.

The concentrations of all the compounds (*C*) were determined from the appropriate optical absorptions (*A*), using the Lambert-Beer law:

$$C=\frac{A}{\varepsilon l}$$ (Eq. 1)

The molar absorption coefficients were *ε*=4.55×10^4^ M^-1^cm^-1^ at *λ*=280 nm for BSA in water (19), *ε*=1.3×10^5^ M^-1^cm^-1^ for PpIXs and *ε*=1.1×10^5^ M^-1^cm^-1^ for PpIXe, both at *λ*=402 nm in DMSO/ACN mixture (both determined in this study). The optical pass was 1 cm.

The experimental data were treated using OriginPro-8 (USA) commercial program and the specialized F9000 software (Edinburgh). All final characteristics are average values of three independent experiments. All experiments were carried out at room temperature (24±1°C).

## Results and Discussion

### Effect of pH on PpIXs characteristics in homogeneous solutions

**Figure 2:**
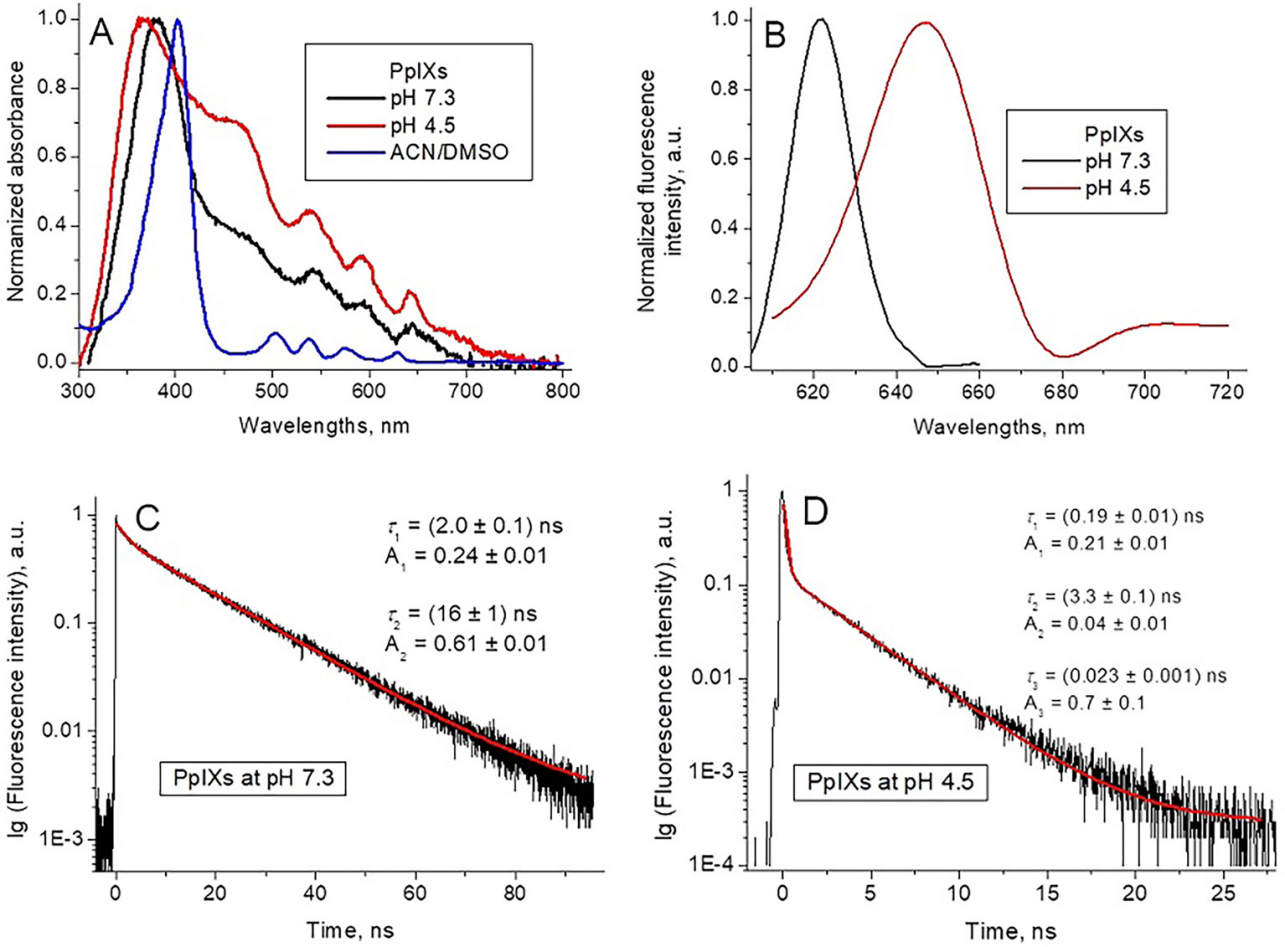
Normalized optical absorption (**A**) and fluorescence (λex=420 nm) (**B**) spectra of synthetic protoporphyrin IX (PpIXs) in different solutions. PpIXs fluorescence decay kinetic curves and respective fittings at pH 7.3 (**C**) and pH 4.5 (**D**) (λex=420 nm, λem=628 nm).

The absorption spectra of PpIXs in DMSO/ACN solutions (Figure 2A) were typical for monomeric forms of free base porphyrins (31), with an intense Soret absorption peak (*λ*max=402 nm) and four peaks with a lower intensity in the Q spectral region.

The absorption spectra of PpIXs in the buffer solution at pH 7.3 (Figure 2A) was diffuse with an absorption maximum at λmax=380 nm and a shoulder in the 440-510 nm range. The Q region displayed only three peaks, shifted to the red spectral region. At pH 4.5 (Figure 2A), the PpIXs largest absorption peak was shifted to 369 nm and the shoulder was transformed to a peak at 463 nm, the positions of three peaks in the Q region were unchanged.

The solubility of PpIXs in water was very low with Csat ≅ 3.9×10-5 M (31). The net charge of PpIXs in pure water was 2^−^. However, in spite of electrostatic repulsion between the molecules, PpIXs forms aggregates in buffer solutions (32). For H-aggregates, a hypsochromic shift of the absorption peaks is characteristic, while the batochromic one is due to formation of J-aggregates (27) and references therein). Analyzing the PpIXs absorption spectra in pure water solutions, the authors in the previous study (32) have proposed the formation of both H- and J-aggregates in an equilibrium.

The pKa of PpIXs is 4.94 (33). There are three points of possible protonation in the PpIXs structure. However, the porphyrin ring structure is the main factor, which determines the absorption and fluorescence spectra of porphyrins. Therefore, the position of maxima in the optical absorption and fluorescence spectra just slightly depends on protonation of collateral groups, while protonation of nitrogen atoms in the porphyrin ring strongly affects the position of absorption and fluorescence maxima. In the case of PpIXs, the pH reduction shifts the fluorescence maximum from *λ*_max_=622 nm at pH 7.3 to *λ*_max_=647 nm at pH 4.5 (Figure 2B). Therefore, we have reason to believe that there is protonation of nitrogen atoms in the porphyrin ring in PpIXs.

The peak formation at λ_max_=463 nm and pH 4.5 (which is just a shoulder at pH 7.3) can be associated with the shift of the equilibrium in favor of J-aggregate formation at low pHs. A similar effect has been reported for the protonation of *meso*-tetrakis(p-sulfonato-phenyl) porphyrin (TPPS_4_) (26).

The intensity of PpIXs fluorescence is weak at both pHs, which is characteristic of porphyrin aggregates (26, 28), and references therein). The red shift of the emission peak from *λ*_max_=622 nm at pH 7.3 to *λ*_max_=647 nm at pH 4.5 (Figure 2B) is characteristic of the protonated porphyrin compared with the non-protonated one (24,26).

The PpIXs fluorescence decay kinetic curve at pH 7.3 (Figure 2C) was successfully fitted as bi-exponential:

IpH7,3=I1 exp−tτ1+I2 exp−tτ2 (Eq. 2)

where *I*_1_, *I*_2_, *τ*_1_, and *τ*_2_ are amplitudes and lifetimes of respective components.

The *τ*_1_ and *τ*_2_ values are shown in Table 1.

**Table 1:** The average values of lifetimes (*τ*_i_) of the fluorescence decay components of PpIXs and PpIXe and their relative contents (*A*_1_ and *A*_2_), calculated in accordance with the equation (Eq. 4), at pH 7.3 and pH 4.5

Porphyrin	pH	*τ*_1_ (ns)	*A*_1_	*τ*_2_ (ns)	*A*_2_
PpIXs	7.3	2.0±0.1	0.28±0.01	16±1	0.72±0.01
	4.5	0.19±0.01	0.84±0.01	3.3±0.1	0.16±0.01
PpIXe	7.3	-	-	16.7±0.5	1
	4.5	-	-	16.5±0.5	1

Data are reported as means±SD. PpIXs: synthetic protoporphyrin IX; PpIXe: endogenous protoporphyrin IX.

It has been demonstrated that aggregation reduces dramatically the lifetimes of porphyrin excited states, both singlet and triplet (28). This effect is typical for various porphyrins and other organic photosensitizers. Thus, we have reason to suppose that the observed short-lived component of the PpIXs fluorescence at pH 4.5 is associated with the fluorescence of its aggregates, while the long-lived one is due to fluorescence of the porphyrin monomers, present in the solution in equilibrium with aggregates. The monomer concentration is much lower than that of the aggregates. However, the quantum yield of the monomer fluorescence generally far surpasses that of the aggregates. Therefore, the amplitude of the long-lived (monomer) fluorescence component (*I*_2_) can be comparable to the amplitude of the fluorescence of aggregates (*I*_1_).

At pH 4.5, the fluorescence decay curve (Figure 2D) was three-exponential:

IpH4,5=I1 exp−tτ1+I2 exp−tτ2+I3 exp−tτ3 (Eq. 3)

Since the lifetime of the third component, *τ*_3_=(0.023±0.005) ns, was close to the exciting pulse duration, this component may be associated with light scattered by aggregates. Protonation reduced the net charge of PpIXs from 2^−^ to 1^−^, thus reducing electrostatic repulsion between the porphyrin molecules, which stimulates their aggregation and increases the aggregate concentrations and sizes. Therefore, at pH 4.5 the scattered light intensity should be higher than that at pH 7.3 and may become significant.

Similar to pH 7.3, at pH 4.5 we associated the first (short-lived) component with fluorescence of aggregates and the second one (long-lived) with fluorescence of monomers. The higher the aggregate concentration, the higher should be the contribution of their fluorescence component (*A*_1_) to the decay curve, calculated as:

$$A_{i}=\frac{I_{i}}{\sum I_{i}}i=1,2$$ (Eq. 4)

where *I*_i_ are the amplitudes of components.

The increase of the contribution of the short-lived component *A*_1_ associated with the porphyrin aggregates was observed at pH 4.5 (Table 1).

In addition, both short-lived and long-lived PpIXs fluorescence components at pH 4.5 were shorter than the respective ones at pH 7.3 (Table 1). The reduction of the fluorescence lifetime due to the porphyrin protonation has been observed for TPPS_4_ porphyrin as well (24). Thus, this result confirmed our hypothesis of PpIXs protonation at pH 4.5.

### Effect of pH on PpIXe characteristics in homogeneous solutions

**Figure 3:**
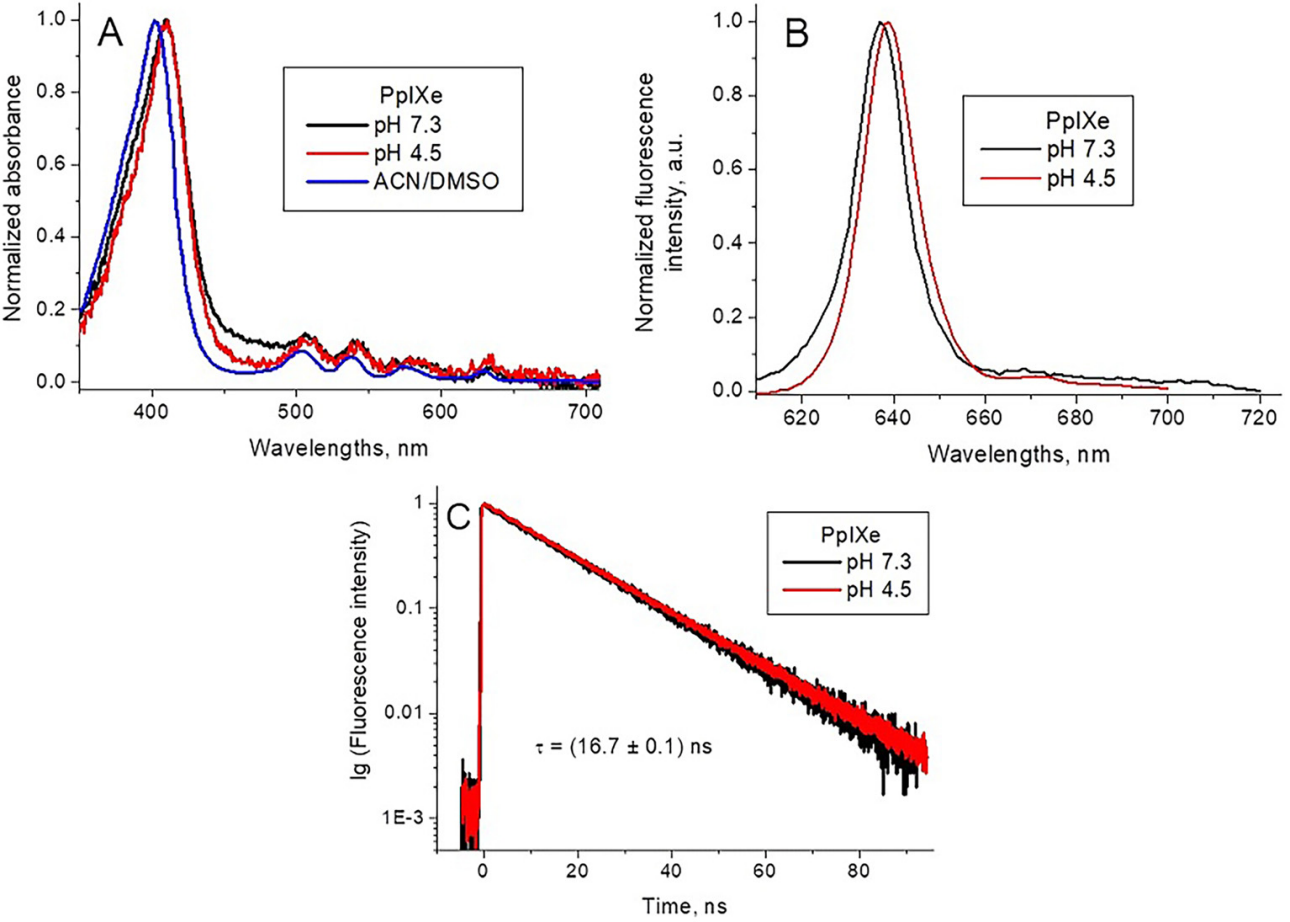
Normalized optical absorption (**A**) and fluorescence (λex=420 nm) (**B**) spectra of endogenous protoporphyrin (PpIXe) in different solutions. PpIXe fluorescence decay kinetic curves and respective fittings at pH 7.3 and pH 4.5 (**C**) (λex=420 nm, λem=637 nm).

Differently from PpIXs, the absorption spectra of PpIXe, both at pH 7.3 and pH 4.5, and in DMSO/ACN solution were similar (Figure 3A), thus evidencing the absence of the porphyrin aggregation. In addition, the change of pH induced no spectral changes, characteristic of porphyrin protonation (24,26). The insignificant red shift of Soret absorption peak may be due to changes in the environment of the molecule.

The PpIXe fluorescence spectra at both pHs were very similar (Figure 3B), with a profile typical of porphyrin monomers (24,26). A weak red shift of the fluorescence peak from 637 nm at pH 7.3 to 639 nm at pH 4.5 may be due to different salt composition of buffer solutions (33).

The fluorescence decay curves at both pHs (Figure 3C) were monoexponential with identical lifetimes. The fluorescence lifetime was similar to that of the long-lived component of the PpIXs fluorescence decay at pH 7.3 (Table 1), which is associated with the fluorescence decay of non-protonated PpIXs monomers. Thus, we can conclude that at both pHs PpIXe was present in the solution as a non-protonated monomer.

**Figure 4:**
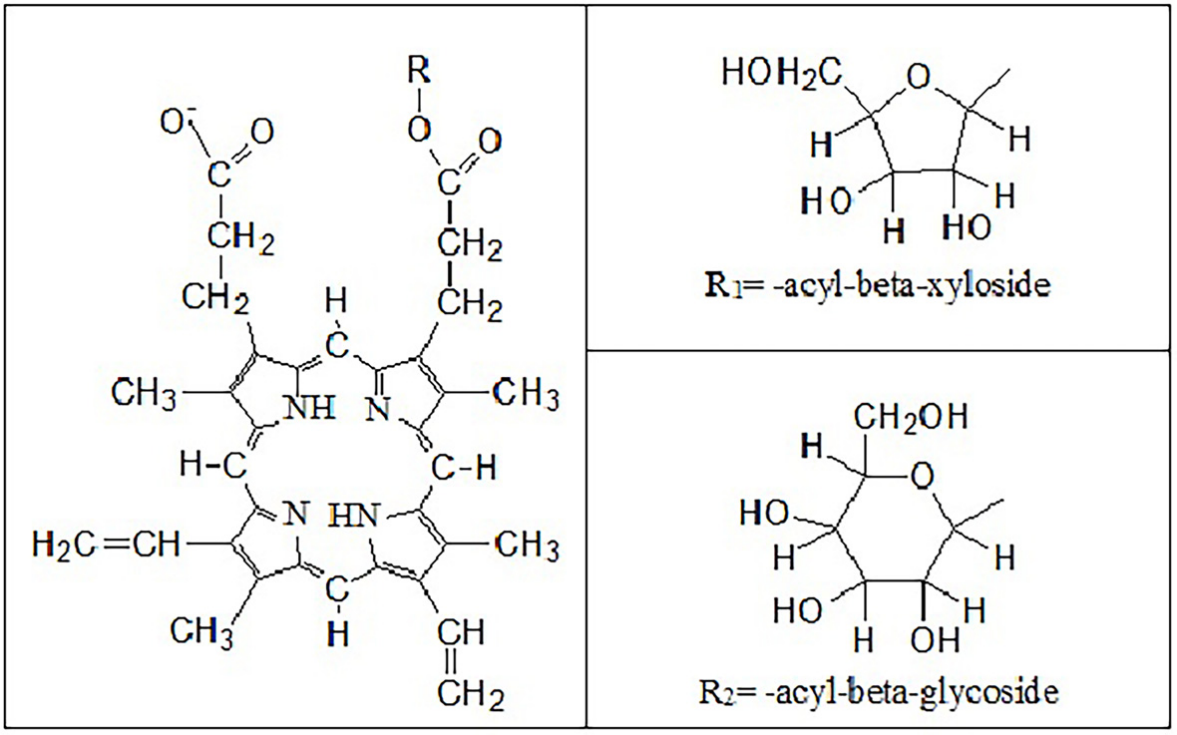
Chemical structures of protoporphyrin-1-O-acyl β-xyloside and protoporphyrin-1-O-acyl β-glucoside.

The composition of PpIXe, extracted from Harderian glands of *ssp Rattus norvegicus albinus* rats, was determined using HPLC, capillary electrophoresis, HPLC/electrospray ionization mass spectrometry (MS), thin layer chromatography, and mass spectrometry (29,30). It was shown that PpIXe consisted of similar amounts of PpIX and its PpIX-1-*O*-acyl β-xyloside derivative with trace quantities of PpIX-1-*O*-acyl β-glucoside (Figure 4).

The authors of a previous study (29) have suggested that conjugation with -acyl-beta-xyloside and/or -acyl-beta-glycoside improves protoporphyrin aqueous solubility. This reduces the porphyrin aggregation. Moreover, we believe that reduction in the net charge of the protoporphyrin-1-*O*-acyl β-xyloside and protoporphyrin-1-*O*-acyl β-glucoside from 2^−^ to 1^−^ should reduce the probability of porphyrin protonation and its pK_a_ point should shift to lower pH values. Hence, at pH 4.5, PpIXe continued to be non-protonated.

### Interaction of PpIXs with BSA

**Figure 5:**
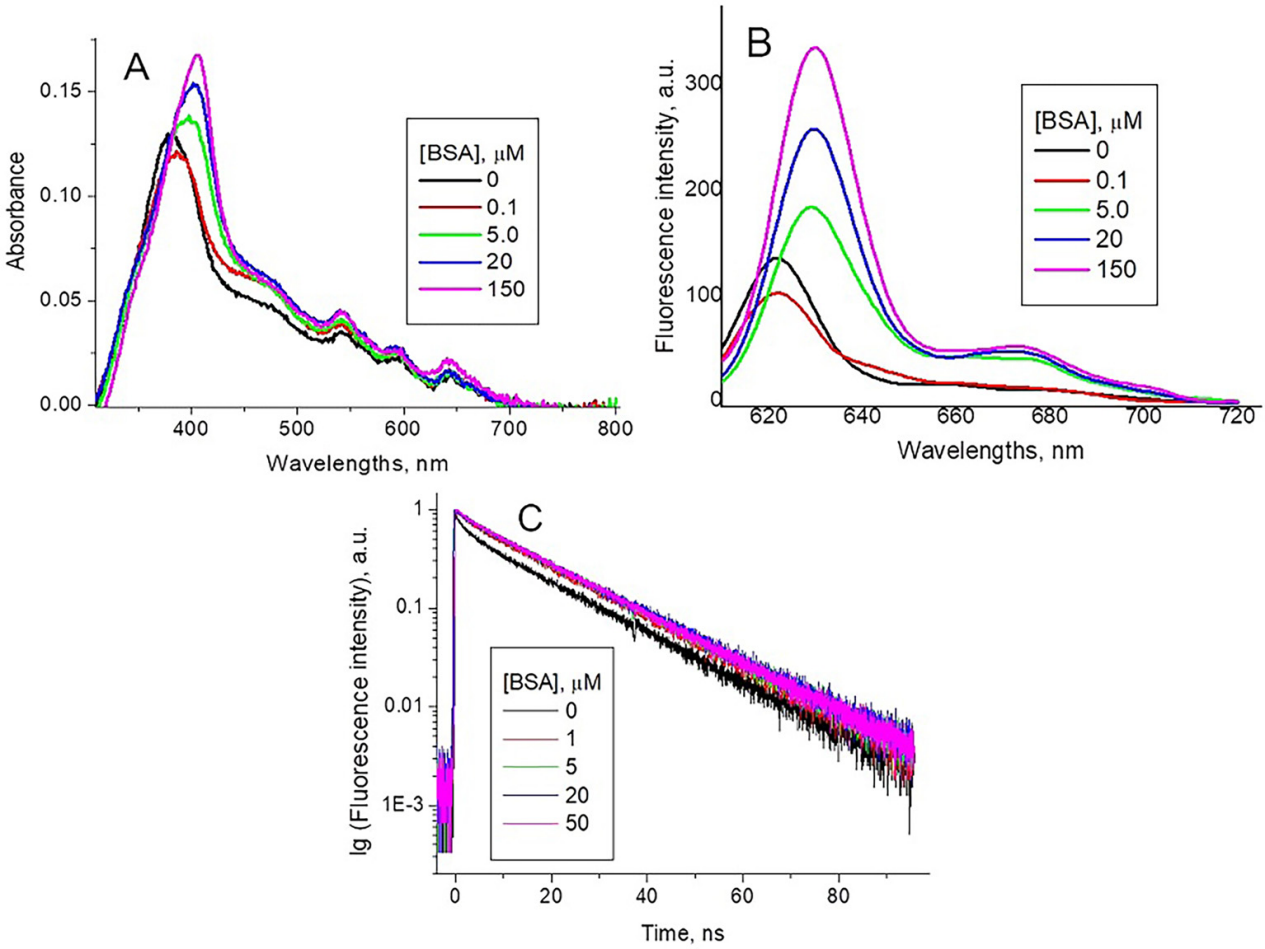
Optical absorption (**A**), fluorescence (**B**) spectra, and fluorescence decay curves (**C**) of 1.3 μM synthetic protoporphyrin IX (PpIXs) at pH 7.3 for different bovine serum albumin (BSA) concentrations (λex=420 nm, λem=628 nm).

In the presence of BSA in concentrations below 0.1 µM at pH 7.3, we observed reduction in the intensity of the PpIXs Soret peak accompanied by a weak red shift from 380 to 382 nm (Figure 5A). Increase in the BSA concentration induced an increase in the intensity of the Soret absorption peak and its shift to 407 nm (Figure 5A). The position of this maximum coincided with that of the Soret peak for PpIXe at pH 7.3, which corresponds to the non-protonated porphyrin monomer.

A similar effect was observed in the presence of BSA for the PpIXs fluorescence profile. BSA in concentrations below 0.1 µM reduced the fluorescence intensity, while higher BSA concentrations increased the fluorescence intensity and induced the shift of its maximum from 622 nm to 633 nm (Figure 5B). The position of the maximum was close to the fluorescence peak of PpIXe at pH 7.3, which corresponds to the non-protonated porphyrin monomer.

The PpIXs fluorescence decay curves in the presence of BSA remained bi-exponential (Figure 5C) with lifetimes *τ*_1_=(2.3±0.3 ns) and *τ*_2_=(16.9±0.6 ns) determined as average values for different BSA concentrations (Table 2), the last one coinciding with that of the non-protonated monomer. The contribution of the short-lived component *A*_1_, calculated according to equation 4, decreased, while *A*_2_ of the long-lived one increased with the increase of the BSA concentration (Table 2).

**Table 2:** The average values of lifetimes (*τ*_i_) of the fluorescence decay components of PpIXs and their relative contents (*A*_1_ and *A*_2_), calculated in accordance with the equation (Eq. 4), at pH 7.3 for different BSA concentrations.

BSA (µM)	*τ*_1_ (ns)	*A*_1_	*τ*_2_ (ns)	*A*_2_
0	2.0±0.1	0.28±0.01	16±1	0.72±0.01
0.01	2.0±0.1	0.24±0.01	16±1	0.76±0.01
5.0	2.4±0.1	0.20±0.01	17±1	0.80±0.01
20.0	2.2±0.1	0.16±0.01	17±1	0.84±0.01
50.0	2.7±0.1	0.12±0.01	18±1	0.88±0.01
150.0	1.9±0.1	0.05±0.01	16±1	0.95±0.01

Data are reported as means±SD. BSA: bovine serum albumin.

Since the short-lived component corresponds to the fluorescence of aggregates and the long-lived one to the non-protonated monomer fluorescence, we can conclude that, at pH 7.3 due to interaction of PpIXs with BSA, the relative content of non-protonated PpIXs monomers increased.

The decrease in the porphyrin optical absorption and fluorescence intensities for BSA concentrations below 0.1 µM can be explained by the fact that in low concentrations, BSA stimulates aggregation of photosensitizers, porphyrins in particular, while higher BSA concentrations provoke their disaggregation (18).

**Figure 6:**
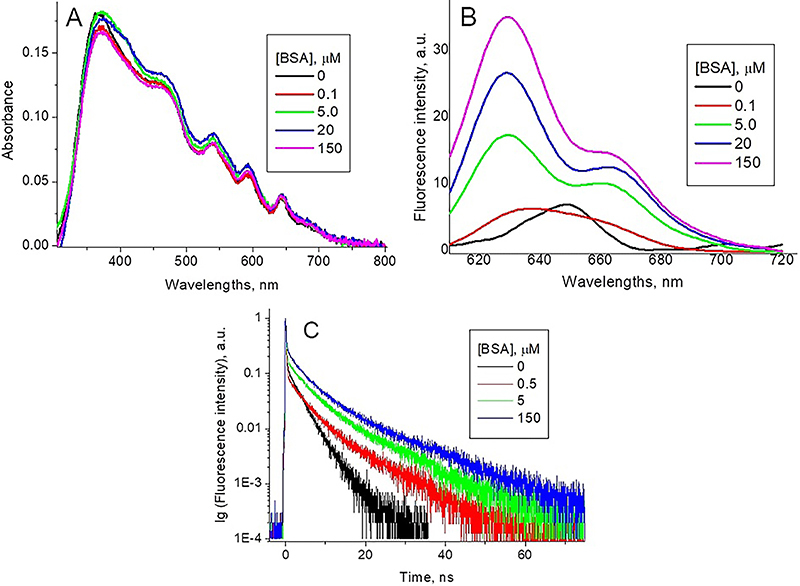
Optical absorption (**A**), fluorescence spectra (**B**) (λex=420 nm), and fluorescence decay curves (**C**) of 1.7 μM synthetic protoporphyrin IX (PpIXs) at pH 4.5 for different bovine serum albumin (BSA) concentrations (λex=420 nm, λem=628 nm).

Addition of BSA to PpIXs solutions at pH 4.5 does not induce any significant changes in the optical absorption spectrum of the porphyrin (Figure 6A). However, the fluorescence peak shifts from *λ*_max_=647 nm in the absence of BSA to *λ*_max_=633 nm after BSA addition (Figure 6B), which is characteristic of fluorescence of the non-protonated porphyrin monomer. A strong increase in the fluorescence intensity was observed as well (Figure 6B).

In the presence of BSA, the fluorescence decay curves transformed to a four-exponential form. However, the shortest component, which lifetime *τ*=(0.05±0.01 ns) was close to the exciting pulse duration, can be due to the scattered light. The other three components with lifetimes *τ*_1_=(0.3±0.1 ns), *τ*_2_=(3.7±0.3 ns), and *τ*_3_=(12±2 ns), determined as average values for different BSA concentrations (Table 3), are attributed to three different porphyrin forms. Relative contributions of these components for different BSA concentrations, calculated in accordance with equation 4, are presented in Table 3.

**Table 3:** The average values of lifetimes (*τ*_i_) of the fluorescence decay components of PpIXs and their relative contents (*A*_1_, *A*_2_ and *A*_3_), calculated in accordance with the equation (Eq. 4) at pH 7.3 for different BSA concentrations.

BSA (µM)	*τ*_1_ (ns)	*A*_1_	*τ*_2_ (ns)	*A*_2_	*τ*_3_ (ns)	*A*_3_
0.5	0.30±0.03	0.76±0.01	3.8±0.3	0.18±0.01	12±2	0.05±0.01
1	0.33±0.05	0.68±0.01	3.3±0.5	0.20±0.01	8±3	0.07±0.05
5	0.37±0.03	0.65±0.05	3.8±0.3	0.31±0.01	12±2	0.09±0.01
20	0.27±0.05	0.63±0.01	3.7±0.4	0.27±0.04	12±2	0.10±0.01
150	0.42±0.05	0.54±0.01	3.9±0.6	0.33±0.01	14±1	0.12±0.01

Data are reported as means±SD. BSA: bovine serum albumin.

The lifetime of the first component was close to that of the protonated PpIXs aggregates. Its contribution *A*_1_ decreased with the increase of BSA concentration. The lifetime of the second one was close to that of the non-protonated PpIXs aggregates (Table 1) and its contribution *A*_2_ increased with BSA concentration. The third component was close to the fluorescence lifetime of the non-protonated PpIXs monomers (Table 1). Its relative contribution *A*_3_ increased with BSA concentration, as well. Thus, we can associate the first component with fluorescence of free protonated PpIXs aggregates, the second with fluorescence of non-protonated porphyrin aggregates bound with BSA, and the third one with fluorescence of non-protonated PpIXs monomers bound with BSA. The dependence of the fluorescence spectrum and the profile of the fluorescence decay curves on BSA concentration showed that BSA binds both PpIXs aggregates and monomers. Moreover, binding with BSA at pH 4.5 stimulated deprotonation of PpIXs molecules both in their monomeric form and in aggregates. This effect can be explained by the fact that the BSA isoelectric point is localized at pH 4.7 (14) and at pH 4.5 its net charge is positive. Therefore, PpIXs monomers and aggregates, bound with BSA, are in the environment with low local proton concentration, which stimulates their deprotonation. Similar effects were observed formerly for the protonated TPPS4 porphyrin (18) and dipyridamole (34).

### Interaction of PpIXe with BSA

**Figure 7:**
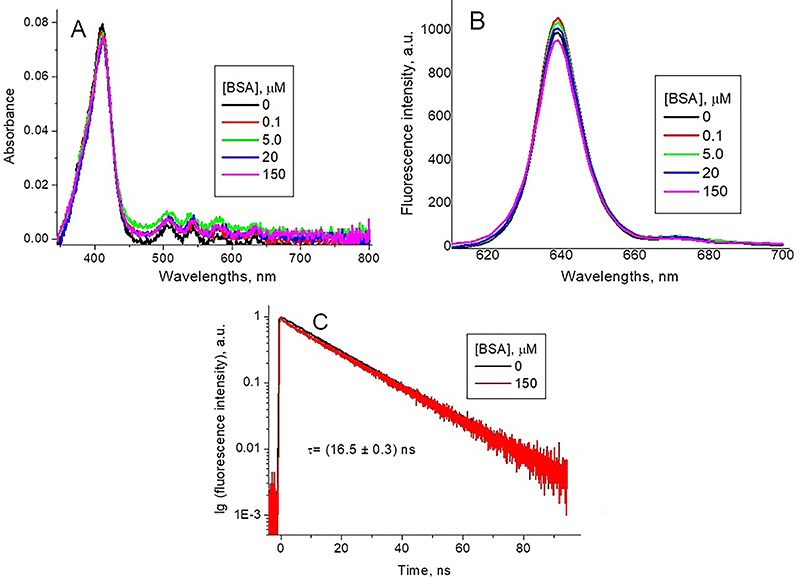
Optical absorption (**A**), fluorescence spectra (**B**) (λex=409 nm), and fluorescence decay curves (**C**) (λex=420 nm, λem=637 nm) of endogenous protoporphyrin (PpIXe) at pH 4.5 for different bovine serum albumin (BSA) concentrations.

Addition of BSA to PpIXe solutions at both pHs produced no significant effect on the porphyrin optical absorption and fluorescence spectra (Figure 7A and B). The profile of fluorescence decay curves continued monoexponential with lifetime *τ*=(16±1 ns). Corresponding data for pH 4.5, as an example, are shown in Figure 7A-C.

Based on the present data, we could expect that PpIXe does not interact with BSA. However, the study of the BSA fluorescence quenching by porphyrins has demonstrated that this assumption was erroneous.

### Quenching of BSA fluorescence by PpIX

**Figure 8:**
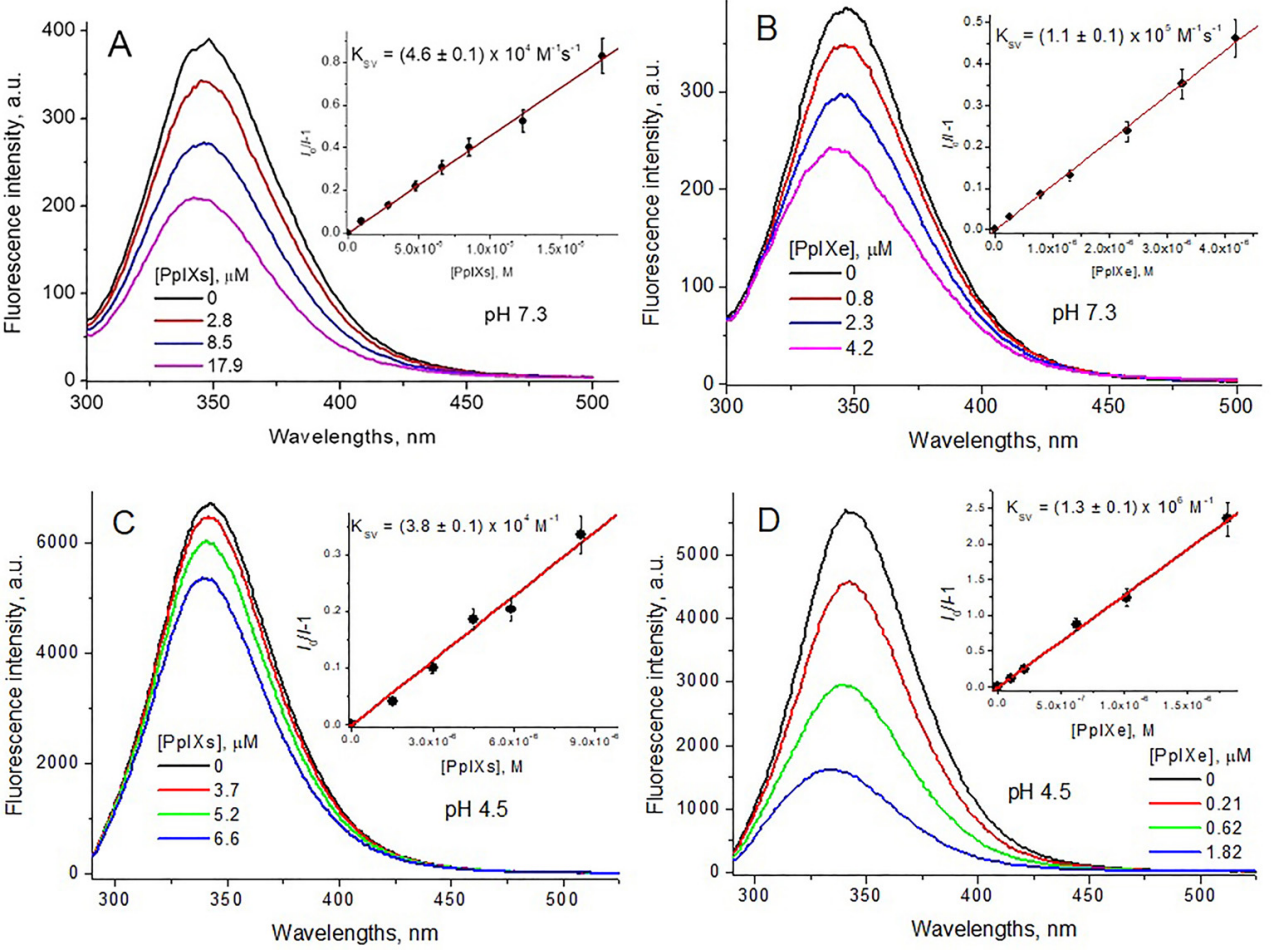
Fluorescence spectra of 8.5 μM bovine serum albumin (BSA) solutions (λex=280 nm) at pH 7.3 (**A**, **B**) and pH 4.5 (**C**, **D**) at different synthetic protoporphyrin IX (PpIXs) (**A**, **C**) and endogenous protoporphyrin IX PpIXe (**B**, **D**) concentrations. Insets are their fittings in accordance with equation 5.

The addition of PpIXs and PpIXe at both pHs decreased the BSA fluorescence intensity (quenching) (Figure 8). The values of the Stern-Volmer quenching constants, *K*_SV_, (Table 4) were determined in accordance with the Stern-Volmer equation (35). To exclude the effect of absorption on the exciting and emitted light (inner filter effect) the *K _SV_* values were corrected in accordance with equation 5 (36):

$$\left(\frac{I_{0}}{I}\right)\eta =1+K_{SV}\left[\text{PpIX}\right]$$ (Eq. 5)

where *I*_0_ and *I* are integral fluorescence intensities in the absence and presence of the quencher in the concentration [PpIX], respectively, and *η* is the correction coefficient, calculated as (36):

$$\eta =\frac{A_{x_{0}}A_{y_{0}}(1-10^{-A_{x_{i}}})(1-10^{-A_{y_{i}}})}{A_{x_{i}}A_{y_{i}}(1-10^{-A_{x_{0}}})(1-10^{-A_{y_{0}}})}$$ (Eq. 6)

where $A_{x_{0}}$ and $A_{y_{0}}$ are the BSA absorbances at the exciting light wavelength and at the emission wavelength; $A_{x_{i}}$ and $A_{y_{i}}$ are the BSA + PpIX absorbances at the exciting light wavelength and at the emission wavelength, respectively.

**Table 4:** The average values of determined (*K*_SV_) and corrected (*K*_SVc_) Stern-Volmer and bimolecular quenching (*k*_q_) constants of the BSA fluorescence quenching by PpIXs and PpIXe at two pHs.

pH	Porphyrin	*K*_SV_ (M^-1^)	*K*_SVc_ (M^-1^)	*k*_q_ (M^-1^s^-1^)
7.3	PpIXs	(4.6±0.1)×10^4^	(4.4±0.1)×10^4^	4.4×10^12^
	PpIXe	(1.1±0.1)×10^5^	(1.0±0.1)×10^5^	1.0×10^13^
4.5	PpIXs	(3.8±0.1)×10^4^	(3.5±0.1)×10^4^	3.5×10^12^
	PpIXe	(1.3±0.1)×10^6^	(1.3±0.1)×10^6^	1.3×10^14^

Data are reported as means±SD. PpIXs: synthetic protoporphyrin IX; PpIXe: endogenous protoporphyrin IX; *K_SV_*_:_ Stern-Volmer quenching constants; bimolecular quenching constant *k*_q_ was calculated in accordance with the equation (Eq. 7).

Bimolecular quenching constants (Table 4), calculated as

$$k_{q}=\frac{K_{\text{SV}}}{\tau_{fl}}$$ (Eq. 7)

where *τ*_fl_ = 10 ns is the BSA fluorescence lifetime (37), in all the cases were higher than the diffusion-controlled one in water *k*_q diff_ ≅ 10^10^ M^-1^s^-1^ (35). This showed that the quenching mechanism is static and *K*_SV_ can be considered as the binding constant (*K*_SV_ ≡ *K*_b_) (35).

At pH 7.3, the BSA net charge is negative (14) and the charge of PpIXs was 2, while at pH 4.5, the BSA net charge is positive (14) and the charge of PpIXs was 1^−^. Therefore, one should expect that due to electrostatic attraction, *K*_b_ for PpIXs binding to BSA at pH 4.5 should be higher than that at pH 7.3. However, in reality, *K*_b_ at pH 4.5 was approximately 20% lower than that at pH 7.3.

Electrostatic interaction is not the only interaction that determines the porphyrin binding with albumins. In various cases, a hydrophobic interaction, for example, can be dominant. Therefore, lower *K*_b_ at pH 4.5 compared with that at pH 7.3 for PpIXs could be explained by a higher hydrophobic effect at pH 7.3. However, the binding constant of the hydrophilic PpIXe at pH 7.3 was 2.3 times higher than that of the hydrophobic PpIXs. Moreover, the charge of PpIXs at pH 4.5 reduced from 2^−^ to 1^−^ due to its protonation. This increased its hydrophobicity, thus increasing, consequently, its aggregation. However, the *K*_b_ of PpIXs at pH 4.5 was lower than that at pH 7.3, where the porphyrin was less hydrophobic. This might confirm our idea about the principal role of electrostatic interaction at the PpIXs binding with BSA.

To explain the lower PpIXs binding constant at pH 4.5 we should remember that there exists an equilibrium between PpIXs monomers and aggregates in buffer solutions. In the presence of BSA, besides the equilibrium between free PpIXs monomers and aggregates in buffer, there exist equilibria between free monomers and monomers bound to BSA and between free aggregates and those bound to BSA as well. If *K*_b_ for monomers is higher than that for aggregates, the observed *K*_b_ should be lower than the real binding constant for the monomer binding to BSA, as previously observed (18).

This supposition is in accordance with the fact, demonstrated above, that the PpIXs aggregation was higher at pH 4.5 than at pH 7.3. Higher aggregation at pH 4.5 explains the lower binding constant of PpIXs at pH 4.5 compared with that at pH 7.3.

This statement is obvious for PpIXe, whose hydrophobicity does not vary with pH changes. PpIXe, with charge 1^−^ at both pHs, is highly water-soluble and does not form aggregates. Thus, the observed *K*_b_ for PpIXe should be its real binding constant to BSA. Indeed, at both pHs, *K*_b_ of PpIXe exceeded that of PpIXs. The *K*_b_ of PpIXe at pH 4.5 was almost 13 times higher than that at pH 7.3. This is due to the fact that at pH 7.3 there exists electrostatic repulsion between the BSA and porphyrin molecules, both negatively charged, while at pH 4.5 the repulsion is replaced by electrostatic attraction between the positively charged BSA and the negatively charged PpIXe, thus demonstrating the principal role of electrostatic effects in the interaction of PpIXe with BSA.

In any case, to confirm these considerations, it is necessary to perform a more profound study to determine the binding sites for both porphyrins in the albumin structure.

### Conclusions

Due to low solubility, PpIXs is present in water solutions in two aggregate forms, H and J, which are in equilibrium with themselves and with monomers. At pH 7.3, the PpIXs charge was 2^−^, while at pH 4.5 it was protonated and had the charge 1^−^. This increased the probability of aggregation and shifted the equilibrium in favor of J-aggregates. The PpIXs interaction with BSA was modulated by PpIXs aggregation. Thus, notwithstanding electrostatic attraction at pH 4.5, where BSA possesses a positive net charge, the binding constant of PpIXs to BSA (*K*_b_) was less than that at pH 7.3, where BSA is negatively charged, and where electrostatic repulsion between BSA and PpIXs molecules exists. The effect can be explained by the fact that *K*_b_ of aggregates to BSA was lower than that of monomers, and the observed *K*_b_ values for PpIXs were less than the real *K*_b_ value for PpIXs monomers. At pH 4.5, PpIXs aggregation was higher than at pH 7.3, which compensated the increase of *K*_b_ due to electrostatic attraction and reduced the observed K_b_ value compared with that at pH 7.3. Binding to BSA at pH 4.5 stimulated deprotonation of PpIXs aggregates and monomers.

Different from PpIXs, PpIXe exists as PpIX-1-*O*-acyl β-xyloside or PpIX-1-*O*-acyl β-glucoside derivatives. It is highly water soluble, and in aqueous solutions exists in the non-protonated monomeric form with the charge 1^−^. Therefore, the obtained *K*_b_ value was the real one for the PpIXe monomers. At both pHs, *K*_b_ of PpIXe exceeded that of PpIXs and at pH 4.5 it was higher than that at pH 7.3 due to electrostatic attraction between the positively charged BSA and negatively charged PpIXe molecules.

The higher probability of PpIXe binding to BSA as compared with that of PpIXs makes PpIXe more promising for use as a fluorescence probe in fluorescence diagnostics and as a photosensitizer for photodynamic therapy. The existence of PpIXe in the monomeric form can explain its faster internalization into the cell as compared with PpIXs. Moreover, aggregation reduces quantum yields and lifetimes of the PpIXs excited states. All these facts can explain higher phototoxicity of PpIXe toward malignant cells compared with PpIXs.

Based on the present results, we can conclude that it is possible to obtain a new photosensitizer for photodynamic therapy or fluorescence diagnostics with synthetized PpIX-1-*O*-acyl β-xyloside or PpIX-1-*O*-acyl β-glucoside derivatives of PpIX by chemical or genetic engineering synthesis, whose efficacy far exceeds that of synthetic porphyrin and which successfully competes with other photosensitizers currently used in photomedicine.
